# Species compositions of terrestrial isopods in public parks of a commuter town in Japan

**DOI:** 10.3897/zookeys.801.21875

**Published:** 2018-12-03

**Authors:** Takahiro Nasu, Kana Kitagawa, Shigenori Karasawa

**Affiliations:** 1 Fukuoka University of Education, 1-1 Akamabunkyo-machi, Munakata, Fukuoka 811-4192, Japan Fukuoka University of Education Munakata Japan; 2 Department of Life and Environmental Agricultural Sciences, Faculty of Agriculture, Tottori University, 4-101 Koyama-machi Minami, Tottori, Tottori 680-8553, Japan Tottori University Tottori Japan

**Keywords:** GIS, GLM, landscape environments, local environments, logistic distributions

## Abstract

The species compositions of terrestrial isopod assemblages in 150 public parks in a commuter town in Japan were investigated. Eleven species of terrestrial isopods were present, of which seven were considered native or oriental species, and four were exotic species originally distributed in the Mediterranean and European regions. An exotic species *Armadillidiumvulgare* Latreille, 1804 was found in all parks. Logistic model analysis indicated that the surrounding land use affected the distributions of three native species, *Burmoniscuskathmandius* (Schmalfuss, 1983), *Ligidiumkoreanum* Flasarova, 1972, and *Mongoloniscuskoreanus* Verhoeff, 1930, indicating that landscape properties are important factors that limit the distributions of terrestrial isopods. The present study also showed that the public parks surrounded by forests or semi-natural environments in a commuter town provide habitats for native terrestrial isopods.

## Introduction

Urbanization has rapidly spread throughout the world and has changed species compositions of regions through decreased diversity of native species (McKinney 2008) and increased numbers of exotics and/or generalist species (Niemelä 1999, Kotze et al. 2011), but the patterns of diversity changes are substantially different depending on biological group and climatic region (Faeth et al. 2011). Thus, understanding and protecting the biodiversity of urban regions is a major ecological concern. Many researchers have contributed to our knowledge of urban biodiversity in recent decades (e.g., McKinney 2008, Jones and Leather 2012, Nielsen et al. 2014), and some suggest that terrestrial isopods are the dominant macro-arthropods in soils of urban regions (Bolger et al. 2000, Smith et al. 2006, Magura et al. 2008a, b, Lee and Kwon 2015). Moreover, it has been observed that urban green spaces in parks provided important habitats that conserve biodiversity in urban environments (Kotze et al. 2011, Barratt et al. 2015). In Japan, many researchers have studied diversity of vegetation, birds, and insects in public parks (e.g., Ishii et al. 1991, Hata et al. 2003, Imai and Nakashizuka 2010, Hattori 2015). However, there has been no comprehensive study of the terrestrial isopods in public parks of Japan.

The Mediterranean region is considered a hotspot of terrestrial isopod diversity (Sfenthourakis and Taiti 2015) and some species that were originally distributed in the Mediterranean and European regions have become dispersed as exotic species throughout the world (e.g., Schmalfuss 2003, Cochard et al. 2010, Hornung et al. 2015). In Japan, seven species that are considered exotic have distribution areas with a potential for expansion (Nunomura 2007, Karasawa and Nakata 2018). These facts suggest that exotic species might already have occupied urban habitats and displaced native species in Japan.

The aims of this study were: 1) to describe the terrestrial isopod fauna in public parks of a commuter town in Japan; 2) to evaluate environments that limit species distributions; and 3) to determine whether public parks in the town provide habitats for native terrestrial isopods.

## Materials and methods

### Study area and sampling method

**Figure 1.**
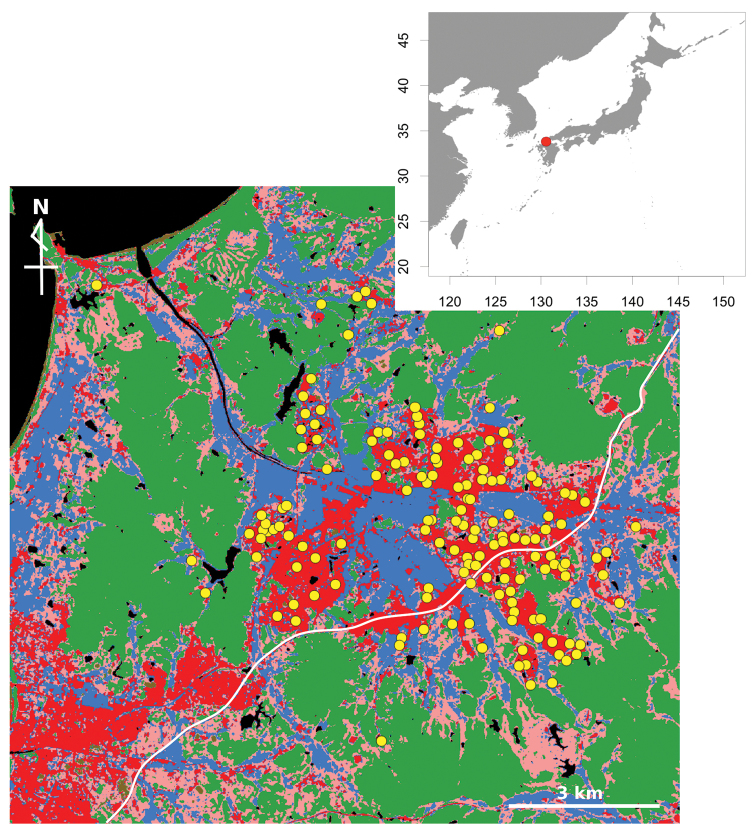
Location of Munakata city (red circle in the top right map) and map of the study area. Yellow circles represent sampling sites (parks). Blue denotes rice paddy; red denotes residential region; pink denotes grass; green denotes forest; the white line is Japan National Route 3. The map was created by modifying the high resolution land-use and land-cover map ver16.02 (http://www.eorc.jaxa.jp/ALOS/lulc/jlulc_jpn.htm).

One hundred fifty public parks were selected in Munakata City, Fukuoka, Japan (Figure 1). Munakata City has been urbanized as a commuter town over several decades. The areas of the parks studied ranged from 74 m^2^ to 17,089 m^2^. Two researchers walked around each park and searched for sites where terrestrial isopods were distributed, before collecting animals. To equalize the sampling effort among parks, the total time for animal collection was standardized as 7 min 30 s. All specimens found were collected, except for the highly abundant *Armadillidiumvulgare* Latreille, 1804, for which only three or four voucher specimens were collected. Sampling was carried out from 27 March to 27 August 2015. In addition, species lists were constructed for forests and a grassland area of the same city to determine the possible source of species in parks. To develop the species list for the forests, five sites (diameter ca. 5 m) were set up inside and at the edge of 18 forests (i.e., 10 sites per forest). In the survey, one researcher collected all terrestrial isopods for 10 min at each site. The species list for grassland was based on that of Tanaka and Karasawa (2018), which was carried out on a grass field in the same city.

Environmental variables were recorded at two levels: local and landscape scales. For local environments, the presence or absence of flower beds and woods in the parks were recorded during sampling. In addition, the percentage of the area of grass in parks was estimated using Google Map (https://www.google.co.jp/maps). For landscape environments, the areas of land use around the parks were measured using geographical information system (GIS) data. A raster data set for a high-resolution land-use and land-cover map (ver. 16.02) was obtained from the advanced land-observing satellite (http://www.eorc.jaxa.jp/ALOS/lulc/jlulc_jpn.htm; Hashimoto et al. 2014). For categorizing land use, grassland and crops were combined as ‘grass’, and four types of forest (broad-leaved, deciduous, evergreen and conifer) were combined as ‘forest’. Thus, four types of landscape environments were considered: ‘rice paddy’, ‘residential region’, ‘grass’, and ‘forest’. To evaluate suitable areas for analysis of landscape environments, buffer zones at five scales were created around each park (widths 50 m, 100 m, 200 m, 500 m, and 1000 m) and the areas of land use in each of the buffers were measured. GIS analyses were carried out using QGIS 2.14 (QGIS Development Team 2009).

### Statistical analyses

To evaluate the appropriate areas to use for the landscape environments, the Akaike information criteria (AIC) of the generalized linear models (GLMs) were compared at the five buffer widths for each species. The GLM was also used to evaluate relationships between the presence of 10 species and the species richness of native and exotic species (as response variables) with respect to environmental variables (as explanatory variables). Logistic (binary) and Poisson distributions were applied to the response variables of presence and species richness (of native and exotic species), respectively. Environmental variables were composed of three local variables (area percentage of grass, presence of flower beds and woods), and four landscape variables (areas of rice paddy, residential region, grass and forest). Using this procedure, perfect or quasi-perfect separations were found in the logistic models for some species. Thus, Firth’s bias-reduced logistic regression (Firth 1993; Heinze and Schemper 2002) was applied to the analysis of the presence of species. The proposed models were compared with the null model by the Likelihood Ratio Test. Significances of coefficient values in the proposed models were evaluated using the Chi-squared test (for presence of species) and the Wald test (for species richness). Multicollinearity among explanatory variables was assessed using variance inflation factors (VIF) for each model: variables with values > 10 were excluded. In addition, spatial autocorrelation of each species and species richness was evaluated by Moran’s *I.* To calculate the Moran’s *I*, distance weight was based on the distances among sampling sites: pairs of sites that are close together have higher values than pairs of sites that are far apart. All statistical analyses were carried out using the functions glm, logistf, vif, lrtest, and Moran.I in R version 3.4.1 (R Core Team 2017). *Armadillidiumvulgare* was excluded from the latter analysis because this species was present in all parks (see Results section).

## Results

**Figure 2.**
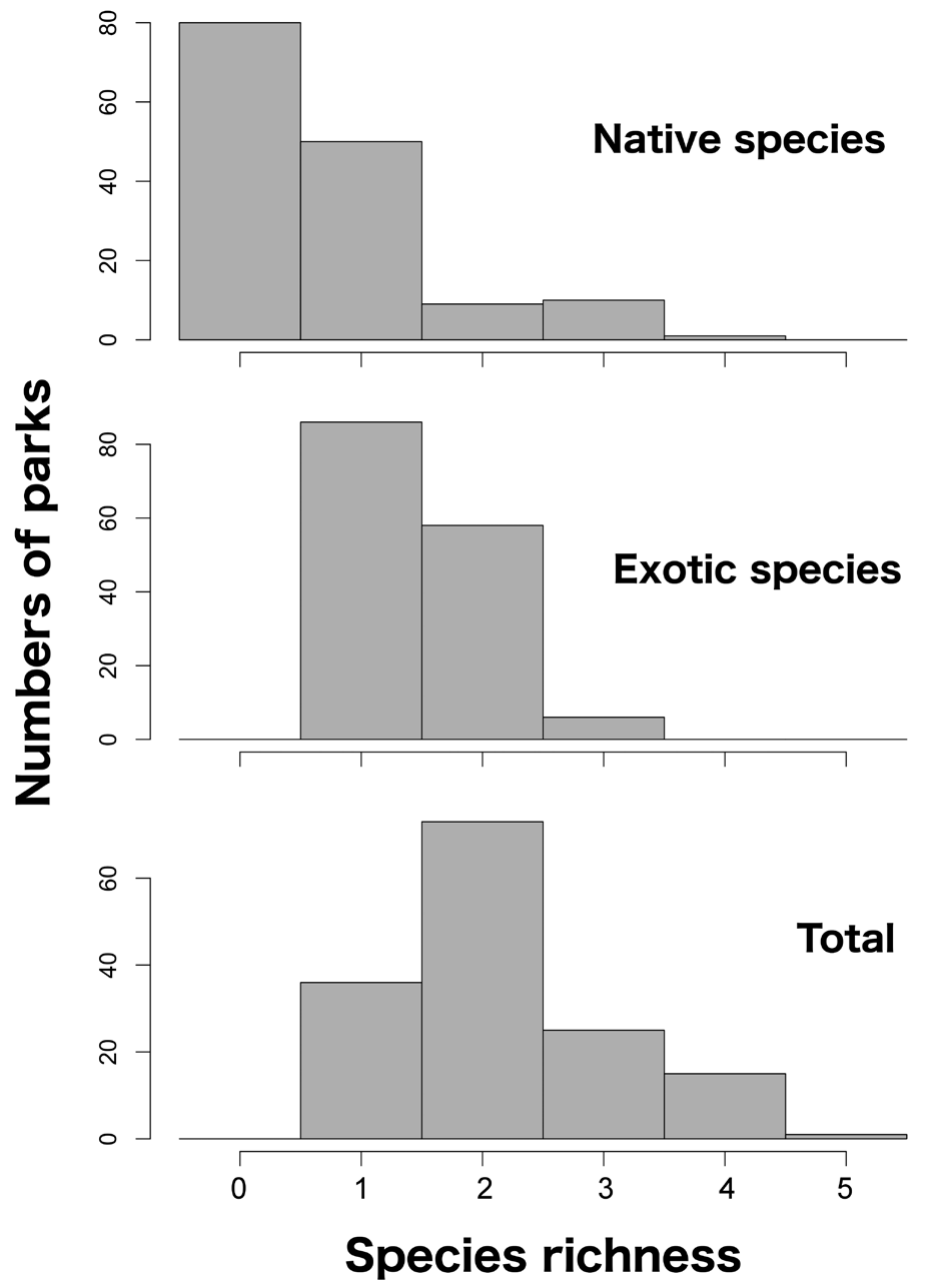
Frequencies of species richness of native, exotic, and total species in parks

A total of 17 isopod species was recorded in the city, eleven of which were collected in the public parks. Five species, *A.vulgare*, *Haplophthalmusdanicus* Budde-Lund, 1880, *Porcelliolaevis* Latreille, 1804, *P.scaber* Latreille, 1804 and *Porcellionidespruinosus* (Brandt, 1833), are exotic species in Japan (Karasawa and Nakata 2018). *Armadillidiumvulgare* was found in all parks and has also become the dominant species in the forests. Seven native species were collected in the parks but five of them were found in nine or fewer parks. In contrast, *Agnarapannuosa* (Nunomura, 1987), Armadillidae sp. 1, *Ligidiumkoreanum* Flasarova, 1972, *Lucasioides* spp. were abundant in the forests. *Mongoloniscuskoreanus* Verhoeff, 1930, *M.vannamei* (Arcangeli, 1927), *P.laevis*, and *P.scaber* were widely distributed in the parks (> 25 parks) but their abundances were low in the forests. *Mongoloniscuskoreanus* was dominant in grassland, as well as *A.vulgare* (Table 1). Overall, species richness in each park ranged from one to five (Figure 2). At least one exotic species was found in each park, but 80 parks lacked native species. The maximum species richness in a park was four for the native species and three for the exotic species.

**Table 1.** Species, numbers of individuals collected and collection sites in the urban parks, glassland and forest in Munakata City, Japan.

Species	Urban park		Grassland^a^	Forest
	No. of ind.	Sites	No. of ind.	No. of ind.
**Native species**				
* Agnara pannuosa *	18	9	a few^b^	1089
Armadillidae sp. 1^c^	9	5	–	1955
Armadillidae sp. 2	–	–	–	60
Armadillidae sp. 3	–	–	–	1
* Burmoniscus dasystylus *	–	–	–	52
* Burmoniscus kathmandius *	17	5	–	–
* Exalloniscus cortii *	–	–	–	1
* Ligidium koreanum *	8	3	–	683
*Lucasioides* spp.	15	8	–	853
* Mongoloniscus koreanus *	172	36	3781	2
* Mongoloniscus vannamei *	110	36	a few	13
*Mongoloniscus* sp.	–	–	a few	–
**Exotic species**				
* Armadillidium vulgare * ^d^	372	150	4526	2220
* Haplophthalmus danicus *	–	–	–	1
* Porcellio laevis *	60	34	–	–
* Porcellio scaber *	264	26	–	21
* Porcellionides pruinosus *	51	10	–	1

^a^ The data from Tanaka and Karasawa (2018)^b^ This species was identified as Agnaridae in Tanaka and Karasawa (2018)^c^ This species was identified as *Spherillo* sp. in Karasawa et al. (2014)^d^ Three or four specimens only were collected from each park.

Values of Moran’s *I* revealed that distribution of *P.scaber* showed significant spatial autocorrelation (*I* = 0.027, *p* < 0.05), while other species and species richness showed no significant spatial autocorrelation. There were differences among species in the spatial scales having the lowest AIC values: *A.pannuosa*, 25 m; Armadillidae sp. 1, 25 m; *B.kathmandius* (Schmalfuss, 1983), 50 m; *L.koreanum*, 500 m; *Lucasioides* spp., 250 m; *M.koreanus*, 100 m; *M.vannamei*, 250 m; *P.laevis*, 50 m; *P.scaber*, 500 m; *P.pruinosus*, 25 m (Supplementary material 1). In addition, the spatial scale of 100 m showed the lowest AIC for the species richness of native and exotic species. Coefficients of variables for the models fitted are described in Table 2. The likelihood ratio test significantly rejected the null model for *B.kathmandius*, *L.koreanum*, *Lucasioides* spp., and *M.koreanus*, and for the species richness of native species. Conversely, there was no significant difference between the proposal and null models for another species and species richness of exotic species, indicating that their distributions were not explained by the seven environmental variables. Local environment significantly affected the distribution of *Lucasioides* spp. only: the presence of flower beds may have a negative effect on the presence of *Lucasioides* spp. The distributions of four native species, *A.pannuosa*, *B.kathmandius*, *L.koreanum*, and *M.koreanus* were significantly affected by landscape properties, although there was no significant difference between the proposed and null models for *A.pannuosa*. *Agnarapannuosa*, *B.kathmandius*, and *L.koreanum* were negatively affected by the surrounding residential region, while natural or semi-natural environments (rice paddy, grass and forest) had positive effects on the distribution of *M.koreanus*. In addition, the model for the species richness of native species showed significant positive values for grass and forest areas, indicating that species richness of native species was high at sites surrounded by natural conditions. However, the model for *P.scaber* indicated a significant positive value for rice paddy only, although *P.scaber* showed spatial autocorrelation and the null model was not rejected.

**Table 2.** Coefficients of explanatory variables and *p* values of the Likelihood ratio test.

Species	Local environments			Landscape environments				Intercept	Likelihood ratio test *p*–value
	Grass	Flower bed	Wood	Residential	Rice paddy	Grass	Forest		
* Agnara pannuosa *	0.181	1.493	-0.634	-**0.105**	-0.083	–	0.074	-2.098	0.177
Armadillidae sp. 1	1.756	-0.026	-1.340	–	-0.068	0.027	0.194	-3.114	0.399
* Burmoniscus kathmandius *	18.912	-0.472	0.745	-**0.047**	-0.036	–	-0.011	-18.440	**0.045**
* Ligidium koreanum *	-2.809	-1.331	-1.137	-**0.001**	-0.001	0.0001	–	4.447	**0.048**
*Lucasioides* spp.	-0.210	-**1.906**	0.148	–	0.001	0.001	-0.002	-2.262	**0.015**
* Mongoloniscus koreanus *	-0.035	-0.031	0.063	–	**0.009**	**0.012**	**0.011**	-3.367	**0.00001**
* Mongoloniscus vannamei *	0.893	1.103	-0.729	–	-0.001	0.001	-0.0002	-1.919	0.243
* Porcellio laevis *	-0.010	0.250	0.632	–	-0.010	-0.018	-0.036	-1.222	0.158
* Porcellio scaber *	1.200	0.452	0.398	–	-0.0004	**0.0000001**	-0.00002	-2.296	0.272
* Porcellionides pruinosus *	0.102	-0.636	0.961	-0.079	0.032	–	-0.150	-2.481	0.395
Number of species									
Native species	0.374	0.061	0.109	–	0.002	**0.006**	**0.004**	-**1.647**	**0.0002**
Exotic species	0.107	0.079	0.327	–	-0.0001	-0.001	-0.001	0.002	0.906

Bold represents *p* < 0.05 by the Chi-squared test.

## Discussion

Lee and Kwon (2015) reported that terrestrial isopods comprised approximately 90% of ground arthropods in urban parks of Osaka, a Japanese mega-city, but they did not identify them at species level. The present study found a total of eleven terrestrial isopod species in the public parks of a commuter town, four of which were exotic species. At least one terrestrial isopod species was found in the parks and the maximum species richness was five. These results imply that urban parks in Japan provide important habitats for terrestrial isopods, as observed in another country (Giurginca et al. 2017).

The species compositions in patchily distributed areas such as parks are known to be affected by local environments within the areas and by landscape environments around the parks (Jokimäki 1999, Germaine and Wakeling 2001, Smith et al. 2005). This study indicated that landscape environments play a more important role in determination of species distributions than do local environments; three native species (*B.kathmandius*, *L.koreanum* and *M.koreanus*) preferred parks surrounded by forest or semi-natural environments (rice paddy and grass) to parks located in residential regions. *Ligidiumkoreanum* and *M.koreanus* have become abundant in forests and grassland within the city. In addition, *B.kathmandius* is widely distributed in East Asia and some Pacific Islands (Schmalfuss1983, Taiti and Ferrara 1991, Karasawa 2016), and is usually found in grasslands of southern regions in Japan (SK, personal observations). Thus, these species are considered grass or forest specialists that may migrate to parks from surrounding semi-natural environments or forests. This finding suggests a hypothesis that the species richness of native species may increase in the parks surrounded by semi-natural and forest environments. The model analysis supported this hypothesis and these observations also indicate that an understanding of the distribution of terrestrial isopods requires evaluation of the surrounding landscape (Dauber et al. 2005).

Four exotic species with original distributions in the Mediterranean and European regions (Schmalfuss 2003, Cochard et al. 2010) were widely distributed in the public parks of the commuter town, but the present study failed to clarify the environmental factors that determined the distribution of exotic species; the null models were not rejected, and *P.scaber* also tended to be geographically concentrated. Three exotic species, *A.vulgare*, *P.scaber*, and *P.pruinosus*, are considered to be a habitat generalist, an urban specialist and a synanthropic species, respectively (Dangerfield and Telford 1990; Magura et al. 2008b), and they have been commonly found in urban regions (Vilisics et al. 2007, 2012, Vilisics and Hornung 2009, Giurginca et al. 2017). This may explain why human activity plays a more important role than environmental factors for their distributions in the city. Moreover, *A.vulgare* was distributed in all parks. This species is able to walk 24 m d^-1^ and is a potentially able to walk 50 m h^-1^ (Furukawa et al. 2017). This high dispersal ability could facilitate the distribution of *A.vulgare* via roads and residential regions and could be an important factor in the establishment and success of populations over a wide range. The establishment of populations of exotic species in public parks has the potential to be a source of exotic species in the future.

## Conclusions

Eleven terrestrial isopod species were found in the urban parks of a commuter town. Native species tended to be distributed in the parks located adjacent to natural environments, while their distributions had little relationship with the local environments within the parks. It is proposed that the location of parks is an important factor to consider in their design to protect the largest number of species of native terrestrial isopods.

## Supplementary materials

Supplementary material 1 AIC values of GLMs at the five apatial scales Data type: species data This is an open access article distributed under the terms of the Creative Commons Attribution License (CC BY 4.0), which permits unrestricted use, distribution, and reproduction in any medium, provided the original author and source are credited.Takahiro Nasu, Kana Kitagawa, Shigenori Karasawa
